# A preferred sequence for organelle inheritance during polarized cell growth

**DOI:** 10.1242/jcs.258856

**Published:** 2021-11-02

**Authors:** Kathryn W. Li, Michelle S. Lu, Yuichiro Iwamoto, David G. Drubin, Ross T. A. Pedersen

**Affiliations:** Department of Molecular and Cell Biology, University of California, Berkeley, Berkeley, CA 94720, USA

**Keywords:** Budding yeast, Mitosis, Endoplasmic reticulum, Peroxisome, Vacuole, Mitochondria, Nucleus, Organelle segregation

## Abstract

Some organelles cannot be synthesized anew, so they are segregated into daughter cells during cell division. In *Saccharomyces cerevisiae*, daughter cells bud from mother cells and are populated by organelles inherited from the mothers. To determine whether this organelle inheritance occurs in a stereotyped manner, we tracked organelles using fluorescence microscopy. We describe a program for organelle inheritance in budding yeast. The cortical endoplasmic reticulum (ER) and peroxisomes are inherited concomitantly with bud emergence. Next, vacuoles are inherited in small buds, followed closely by mitochondria. Finally, the nucleus and perinuclear ER are inherited when buds have nearly reached their maximal size. Because organelle inheritance timing correlates with bud morphology, which is coupled to the cell cycle, we tested whether disrupting the cell cycle alters organelle inheritance order. By arresting cell cycle progression but allowing continued bud growth, we determined that organelle inheritance still occurs when DNA replication is blocked, and that the general inheritance order is maintained. Thus, organelle inheritance follows a preferred order during polarized cell division and does not require completion of S-phase.

## Abstract (precis)

**Summary:** Organelles are interconnected by contact sites, but they must be inherited from mother cells into buds during budding yeast mitosis. We report that this process occurs in a preferred sequence.

## INTRODUCTION

Cell duplication via polarized cell growth presents a unique challenge to cellular organization. In contrast to isotropic growth – which can occur through expansion of existing cellular structure and organization – during polarized growth that leads to cell duplication, either a new cellular structure must be constructed from scratch, or existing cellular components must be transported and rearranged in a regulated manner. Similarly, during development, neurons grow axons in order to properly wire the nervous system. This growth requires coordinated production and movement of various cellular components, which is regulated by signaling between the end of the growing axon and the cell body (Goldberg, 2003). Defects in organelle positioning within axons have been implicated in various neurological diseases including Charcot–Marie–Tooth disorder (Suárez-Rivero et al., 2017).

Many organelles cannot be readily made *de novo*, and therefore must be trafficked into newly forming cellular structures, such as axons or yeast daughter cells during polarized growth (Nunnari and Walter, 1996; Warren and Wickner, 1996). This process is complicated by the fact that organelles are interconnected through a network of membrane contact sites (Murley and Nunnari, 2016; Wu et al., 2018). These membrane contact sites have been implicated in crucial cellular processes ranging from lipid transfer between organelles to coordination of organelle division (AhYoung et al., 2015; Friedman et al., 2011; Lewis et al., 2016; Maeda et al., 2013). While organelle organization within the cytoplasm is critical for organelle function, how this ordered arrangement is maintained or reestablished as organelles are inherited during polarized cell growth remains a mystery.

To explore how directed movement of organelles is coordinated during polarized cell growth, we studied organelle inheritance in *S. cerevisiae*. This organism reproduces asexually by budding, wherein the daughter cell forms as a ‘bud’ from the mother before being released by cytokinesis at the end of the cell cycle. Organelles and other cellular materials synthesized in the mother cell are actively transported to the growing daughter cell. Numerous studies have investigated the molecular mechanisms that facilitate inheritance of organelles during *S. cerevisiae* bud growth. Most organelles, including endoplasmic reticulum (ER), peroxisomes, mitochondria and vacuoles, are transported into buds by a common mechanism – Myo2, a processive type V myosin motor, binds to organelles via organelle-specific adaptor proteins and walks them along actin cables that extend from the mother cell into the bud (Pruyne et al., 2004; Weisman, 2006). While Myo2 is similarly involved in the early migration of the nucleus to a position close to the bud neck (Yin et al., 2000), movement of the nucleus through the bud neck follows a distinct, microtubule-based mechanism (Huffaker et al., 1988), wherein dynein motors localized to the bud cortex pull the spindle and nucleus through the bud neck (Adames and Cooper, 2000). Despite extensive investigation, organelle inheritance pathways in budding yeast have mostly been studied individually. Therefore, how or whether inheritance of different organelles is coordinated remains largely unexplored.

Recent research hints that organelle inheritance may occur in an ordered manner, despite the common mechanism that governs bud-directed movement of many organelles. One study found that membrane contact sites formed between mitochondria and the plasma membrane of emerging buds double as anchoring sites for dynein motors that move the nucleus into the bud (Kraft and Lackner, 2017). Such a mechanism, wherein inherited mitochondria set up the machinery to ensure nucleus inheritance, suggests a preferred order of organelle inheritance. We wondered whether other organelles, such as those that are transported into buds by a common mechanism, were also inherited in a preferred order.

We performed time-lapse imaging of five organelles during budding yeast mitosis to compare their inheritance. We report a preferred succession of organelles into growing buds that occurs in three stages, beginning with cortical ER and peroxisome inheritance as the bud emerges, followed by vacuole and mitochondria inheritance into small buds, and, finally, ending with nuclear and nuclear ER inheritance into large buds. Surprisingly, cell cycle disruption did not affect organelle inheritance itself, nor the ordering of these three stages. Specifically, blocking S-phase, which normally begins around the time of bud emergence, did not alter the order of these three phases, although the nucleus was not inherited, and the inheritance order of mitochondria and vacuoles was reversed. Our data suggest that interdependent translocation or signaling pathways orthogonal to cell cycle signaling may enforce order on organelle inheritance during *S. cerevisiae* polarized growth.

## RESULTS AND DISCUSSION

To determine whether organelle inheritance follows a stereotyped order during budding yeast mitosis, we studied five organelles using live-cell, 3D time-lapse imaging. For each organelle, the time from bud emergence to organelle inheritance was measured. As established in the classic studies of Hartwell and colleagues (Culotti and Hartwell, 1971; Hartwell, 1971; Hartwell et al., 1970), bud morphology in budding yeast is highly correlated with cell cycle stage. Using bud emergence to define the start of each time course allowed us to compare the inheritance timing of organelles in different cells. We imaged cells using bright field microscopy for various time periods before collecting fluorescence time courses in order to capture both the moment of bud emergence and the organelle inheritance process at high temporal resolution and without significant photobleaching of genetically encoded fluorophores. To mark different organelles, yeast strains endogenously expressing C-terminal GFP fusions of proteins known to localize to organelles of interest were used in most cases. Peroxisomes were visualized via Pex3–GFP (Huh et al., 2003), vacuoles were visualized via Vph1–GFP (Lu and Drubin, 2020), mitochondria were visualized via Cit1–GFP (Sawyer et al., 2019) and nuclei were visualized via Nup59–GFP (Madrid et al., 2006). The ER was visualized by expressing a single copy of GFP–HDEL integrated into the genome at the *TPI1* locus (Lu and Drubin, 2020). While other organelles, including secretory vesicles (Schott et al., 2002) and the Golgi (Arai et al., 2008; Rossanese et al., 2001), are inherited during budding, they are also continuously generated from organelles upstream in the secretory pathway, confounding our analysis. We therefore excluded them from our study. Cells also endogenously expressed mCherry-tagged Myo1, the contractile ring myosin, to clearly delineate the boundary between mother and daughter cells and to mark the onset of cytokinesis, when the ring begins to contract.

**Fig. 1.**
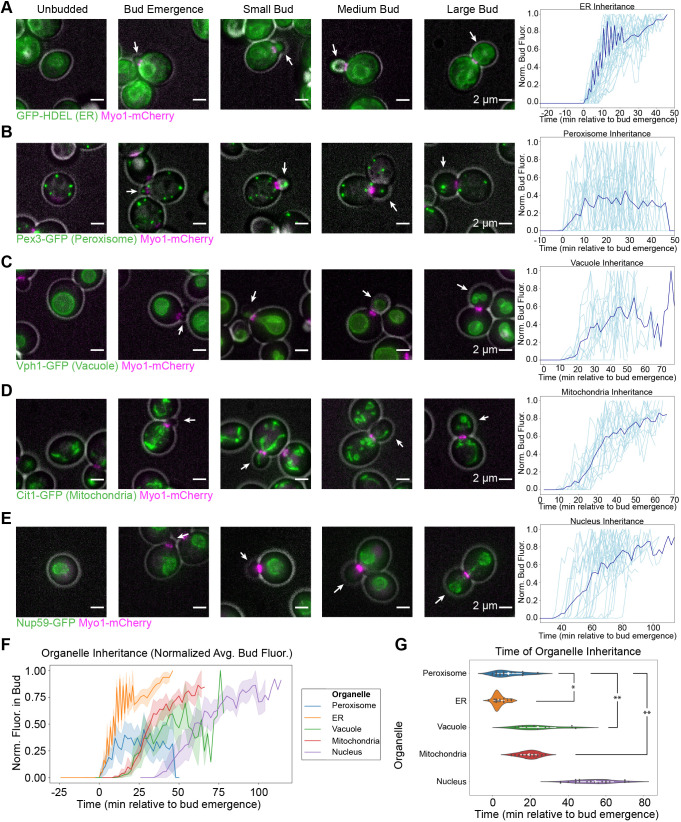
**Organelle inheritance occurs in three distinct stages.** (A) Left, maximum intensity projections from epifluorescence stacks of cells endogenously expressing Myo1–mCherry (magenta) to label the cytokinetic contractile ring and expressing GFP–HDEL to label the ER (green). Gray, cell outline from bright-field imaging. White arrows identify the bud in each frame. Cells at different cell cycle phases are juxtaposed to illustrate succession. Right, normalized GFP–HDEL signal in the bud as a function of time from when bud emergence is detectable, measured from 23-frame, 44-min movies. Dark blue line, mean fluorescence versus time trace from 34 cells from five experiments. Individual measurements shown in light blue. (B–E) Left, maximum intensity projections of cells endogenously expressing Myo1–mCherry (magenta) and Pex3–GFP (green, peroxisomes, B), Vph1–GFP (green, vacuoles, C), Cit1–GFP (green, mitochondria, D) or Nup59–GFP (green, nuclei, E), montaged with gray cell outlines as in A. Right, normalized GFP signal in the bud versus time, measured as in A. Dark blue lines, mean fluorescence versus time traces from 29 cells from six experiments (B), 12 cells from five experiments (C), 17 cells from five experiments (D), and 22 cells from six experiments (E). Individual measurements are shown in light blue. (F) Mean fluorescence (with 95% confidence intervals) versus time traces for organelles imaged in A–E plotted on the same axes for direct comparison. (G) Violin plots for the inheritance times of the organelles imaged in panels A–E with mean values and 95% confidence intervals shown in white and raw data shown as dark gray points. Inheritance time was defined as the first time when the bud fluorescence surpassed 0.5% of the maximum total fluorescence for the peroxisome or 2.5% of the maximum total fluorescence for the other organelles, which approximates the inflection point of the curves. Inheritance times were compared by two-tailed Welch's ANOVA (*F*=165) followed by Games–Howell test. Asterisks indicate statistical significance between organelles whose inheritance time confidence intervals do not overlap. As the 95% confidence interval for nuclear inheritance timing did not overlap with the 95% confidence interval of any other organelle, it was excluded from statistical tests and considered significantly different from all other organelles. *P<0.05 (*P*=0.0193), **P<0.01 (*P*=0.0010 for both comparisons).

Our data indicate that organelle inheritance occurs in three stages. The cortical ER, which lines the cell periphery, and the peroxisomes, are the earliest organelles inherited, with inheritance beginning concomitantly with bud emergence (Fig. 1A,B). Peroxisomes are the most dynamic of the organelles that we imaged, and they became particularly difficult to track as the growing bud got bigger, allowing them more space to dynamically occupy. Nevertheless, they can clearly be seen entering the smallest buds observed (Fig. 1A). Vacuoles and mitochondria are inherited slightly later, in small buds, with inheritance commencing 10–20 min after bud emergence (Fig. 1C,D). Finally, nuclei are inherited once cells have reached the large bud stage, ∼40 min after bud emergence (Fig. 1E). Perinuclear ER, which is continuous with the nuclear envelope, behaves similarly to the nucleus itself (Fig. 1A).

Plotting the average, normalized organelle fluorescence in the bud as a function of time for all five organelles on the same axes clearly reveals the three stages of inheritance, beginning when cortical ER and peroxisomes are inherited, followed by vacuoles and mitochondria, and ending with nuclear inheritance (Fig. 1F). We functionally defined an inheritance event for an organelle as being the timepoint when fluorescence intensity for that organelle accumulated to a threshold percentage of its maximum in the bud. The threshold was defined operationally as a fluorescence intensity past which traces rarely fluctuated back to zero. Directly comparing the timepoint of inheritance for each organelle confirms that peroxisomes and cortical ER are inherited with similar kinetics (Fig. 1G). Our statistical tests even indicated that cortical ER is inherited significantly before peroxisomes, but the difference in timing and *P*-value for this result were each an order of magnitude less than for all other observed differences. Mitochondria and vacuoles are inherited significantly after the peroxisomes and with similar kinetics, consistent with previous findings (Eves et al., 2012). Finally, nuclei are inherited significantly after all other organelles, consistent with previous observations and reflecting their distinct inheritance pathway (Pruyne et al., 2004). While we were able to define these three stages of inheritance by imaging organelles individually and using bud emergence as a common time reference, we were not able to resolve fine-grained differences in the timing of organelle inheritance within each stage by this analysis.

**Fig. 2.**
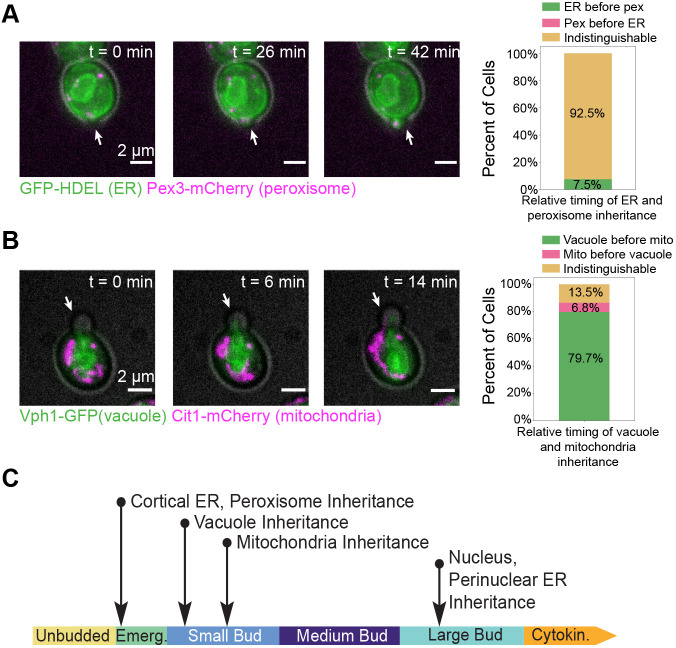
**Direct comparison of inheritance within phases resolves inheritance order to elucidate a timeline.** (A) Left, maximum intensity projections from a 3D time lapse epifluorescence series (26 frames, 25 min total) of a cell expressing GFP–HDEL (green, ER) and endogenously expressing Pex3–mCherry (magenta, peroxisomes). Gray, cell outline from bright-field imaging. Right, percentage of 53 cells from three experiments in which the ER is inherited before the peroxisomes (green bar), peroxisomes are inherited before the ER (magenta bar), or the order is indistinguishable (yellow bar). (B) Left, maximum intensity projections from a 3D time lapse epifluorescence series (26 frames, 25 min total) of a cell endogenously expressing Vph1–GFP (green, vacuole) and Cit1–mCherry (magenta, mitochondria). Gray, cell outline from bright field imaging. Right, percentage of 117 cells from three experiments in which vacuoles are inherited before mitochondria (green bar), mitochondria are inherited before vacuoles (magenta bar), or the order is indistinguishable (yellow bar). White arrows in A and B point to the bud in each frame. (C) A timeline summarizing the observed inheritance timing of organelles during yeast budding.

For organelles whose inheritance timing was indistinguishable using single-color imaging, we imaged pairs of organelles using two-color 3D time-lapse imaging to resolve differences in inheritance timing. Although we occasionally observed that the cortical ER was inherited in emerging buds prior to peroxisomes, the inheritance timing of cortical ER and peroxisomes was still indistinguishable in most cases (Fig. 2A; Movie 1). On the other hand, when we directly compared vacuole inheritance with mitochondrial inheritance, we observed that vacuoles are inherited before mitochondria (Fig. 2B; Movie 2). This finding differs slightly from what was observed in a previous study, which reported that vacuole inheritance precedes mitochondria inheritance only 60% of the time (Eves et al., 2012). One possible explanation for this discrepancy is our use of 3D time lapse imaging, as opposed to imaging only in the medial focal plane of the cells. Taken together, these results define a timeline for organelle inheritance (Fig. 2C). Cortical ER and peroxisomes are inherited immediately upon bud emergence. Next, vacuoles and then the mitochondria are inherited at the small bud stage. Finally, nuclei are inherited at the large bud stage.

We next set out to determine whether organelle inheritance order is coordinated with cell cycle events. Because organelle inheritance events were observed at specific points during bud growth, and because the bud morphogenesis cycle is tightly linked to the cell cycle, we wondered whether disrupting the cell cycle would impact the order of organelle inheritance. To test this possibility, we took advantage of the fact that hydroxyurea effectively halts S-phase in budding yeast without arresting the bud morphogenesis cycle (Alvino et al., 2007; Amberg et al., 2005). Hydroxyurea treatment allowed us to assess how organelles are inherited when the cell cycle is disrupted.

**Fig. 3.**
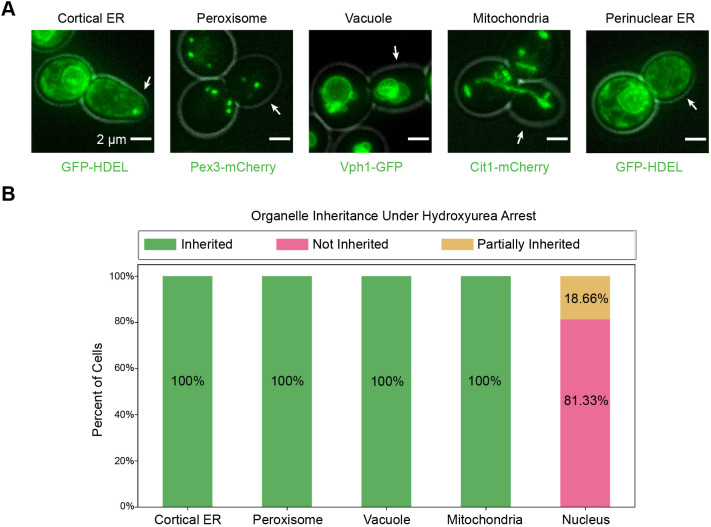
**Organelle inheritance does not require completion of S-phase.** (A) Maximum intensity projections from epifluorescence stacks of hydroxyurea-treated cells. From left to right, cells are expressing GFP–HDEL to visualize the cortical ER, endogenously expressing Pex3–mCherry (peroxisomes), Vph1–GFP (vacuoles), Cit1–mCherry (mitochondria), and GFP–HDEL to visualize the perinuclear ER (all in green). Gray, cell outline from bright field imaging. White arrows point to the bud in each frame. (B) Percentage of cells (*n*=75 cells from three experiments for cortical ER, peroxisome, and perinuclear ER; 80 cells from three experiments for vacuole and mitochondria) in which the organelle of interest was inherited (green bar), not inherited (magenta bar), or partially inherited (yellow bar) in the presence of hydroxyurea.

While we hypothesized that organelle inheritance timing might be controlled in part by the cell cycle, we found that organelle inheritance mostly continues even when S-phase is blocked, consistent with previous studies of peroxisome, cortical ER, and mitochondrial inheritance (Fagarasanu et al., 2005; Loewen et al., 2007; Yang et al., 1999). We arrested cells in hydroxyurea for 3 h, sufficient time for cells that were past S-phase when the drug was added to complete their cell cycle and arrest at the following S-phase, giving us confidence that all cells experienced an S-phase block. After hydroxyurea treatment, cells were morphologically arrested at the large bud stage of the growth cycle, which normally corresponds to late M-phase (Fig. 3A). Even though cortical ER and peroxisomes are normally inherited in emerging buds (around the time of S-phase onset) and all other organelles are inherited in growing buds (after S-phase onset), we nevertheless observed cortical ER, peroxisomes, vacuoles and mitochondria in most of the large buds that had grown from the hydroxyurea-treated cells (Fig. 3A,B). Nuclei, on the other hand, remained either in the mother cell (not inherited) or at the bud neck (partially inherited) (Fig. 3A,B). A two-tailed χ-squared test of these data rejected the null hypothesis that organelles are distributed to the mother, bud neck and bud in proportion to the relative areas of these regions in a two-dimensional maximum intensity projection (χ-squared=134,074 for cortical ER and peroxisomes, 143,215 for vacuoles and mitochondria, 49,291 for nuclei, *P*<0.0001 for all), supporting the conclusion that each organelle is asymmetrically distributed in hydroxyurea-treated cells. Thus, even when S-phase completion is blocked, inheritance of organelles that depends primarily on actin-based transport can proceed.

**Fig. 4.**
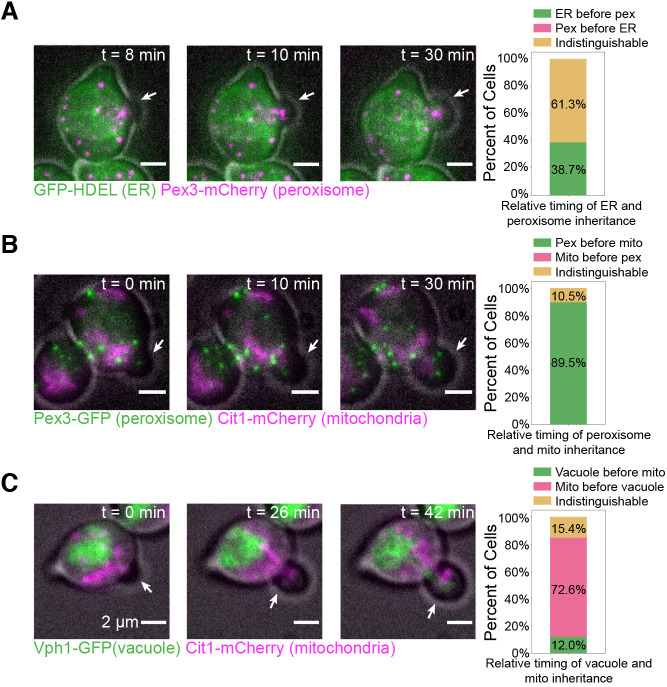
**Order of organelle inheritance remains largely normal when S-phase is not completed.** All images on the left show maximum intensity projections from 3D epifluorescence time lapse series (23 frames, 44 min total) of cells after α-factor synchronization and release into hydroxyurea. Gray, cell outline from bright field imaging. White arrows point to the bud in each frame. (A) Left, a cell expressing GFP–HDEL (green) and endogenously expressing Pex3–mCherry (magenta). Right, percentage of 31 cells from six experiments where the ER is inherited before peroxisomes (green bar), peroxisomes are inherited before the ER (magenta bar), or the exact order is indistinguishable (yellow bar). (B) Left, a cell endogenously expressing Pex3–GFP (green) and Cit1–mCherry (magenta). Right, percentage of 38 cells from three experiments in which peroxisomes are inherited before mitochondria (green bar), mitochondria are inherited before peroxisomes (magenta bar), or the order is indistinguishable (yellow bar). (C) Left, a cell endogenously expressing Vph1–GFP (green) and Cit1–mCherry (magenta). Right, percentage of 38 cells from six experiments in which vacuoles are inherited before mitochondria (green bar), mitochondria are inherited before vacuoles (magenta bar), or the order is indistinguishable (yellow bar).

When we examined organelle inheritance timing, we found that the order of the three stages of inheritance we observed previously remained the same even without continuous cell cycle progression. To study organelle inheritance timing, we used α-factor to synchronize cells in G1, prior to S-phase and bud emergence, and then released them into hydroxyurea for imaging (Amberg et al., 2005). This eliminated the possibility that bud growth observed occurred in cells that were past S-phase at the time of hydroxyurea addition, ensuring that all bud growth observed occurred under hydroxyurea arrest. This procedure allowed us to record time series of organelle inheritance while bud growth was occurring despite cell cycle perturbation. We found that both the cortical ER and peroxisomes were still inherited at bud emergence, with the inheritance timing of these two organelles still mostly indistinguishable (Fig. 4A; Movie 3). As in our earlier results, peroxisomes were clearly inherited before the mitochondria, indicating that the first two stages of organelle inheritance that we had observed were still separable (Fig. 4B; Movie 4). In a departure from our results with unmanipulated cells, we observed the mitochondria being inherited before the vacuole, but both organelles were still inherited into small buds (Fig. 4C; Movie 5). Thus, despite small changes in the order of organelle inheritance within a given stage, such as with the vacuole and mitochondria, the overall order of the different stages remained the same under an S-phase block.

Our results demonstrate that organelle inheritance in budding yeast occurs in a predictable order. Previous studies of the mechanisms underlying organelle inheritance in this organism typically studied organelles individually, going so far as to demonstrate that failed inheritance of one organelle had no major effects on the inheritance of others (see, for example, Du et al., 2001; Ishikawa et al., 2003). More recent studies, however, hint that some organelle inheritance pathways are interdependent (Kraft and Lackner, 2017). The fact that organelle inheritance follows a stereotyped timeline (Fig. 2C) suggests that other such interdependent organelle inheritance pathways may be at play during budding yeast mitosis.

We also found that most organelles are inherited even when S-phase progression is chemically inhibited. Some studies have shown that proteins involved in inheritance of specific organelles are regulated by cell cycle signaling (Fagarasanu et al., 2005; Peng and Weisman, 2008). Our results demonstrate that successful inheritance of the cortical ER, peroxisomes, vacuoles and mitochondria does not require S-phase completion (Fig. 3A,B). Moreover, the coupling of organelle inheritance to bud morphology remains largely unchanged, with organelles being inherited during the same morphological stages as described in our timeline of organelle inheritance for wild-type cells (Fig. 2C). This observation suggests that while cell cycle signaling may influence inheritance of individual organelles, different signaling pathways regulate the relative order in which organelles are inherited.

The observation that inheritance of individual organelles occurs at distinct stages of bud morphogenesis suggests intriguing possibilities regarding mechanisms controlling inheritance timing. Geometric constraints, such as the size of the opening at the bud neck, may play a role in determining the inheritance sequence. Signaling pathways orthogonal to cell cycle signaling may also be at play; a recent study described how non-cell-cycle cues – including signaling by the polarity regulator Cdc42, priming of septins, and cell wall weakening – control the timing of bud emergence (Lai et al., 2018). Furthermore, one study showed that loss of cortical ER inheritance disrupts septin assembly, hinting that organelle inheritance and bud morphogenesis may be interdependent (Loewen et al., 2007). After bud emergence, inheritance of organelles may be governed by interdependent inheritance pathways. These pathways may ensure that organelle–organelle contact sites and their associated inter-organelle functions, such as lipid exchange, are maintained after cytokinesis.

## MATERIALS AND METHODS

### Strains and plasmids

All strains used in this study are listed in Table S1. Budding yeast strains were all derived from wild-type diploid DDY1102 and propagated using standard techniques (Amberg et al., 2005). The GFP-HDEL strain was constructed by integrating a GFP-HDEL::LEU plasmid (courtesy of Laura Lackner, Northwestern University Department of Molecular Biosciences, Evanston, IL, USA) at the *TPI1* locus. This plasmid is the pRS305 backbone containing the *TPI1* promoter, followed by the leader sequence of *KAR2* (amino acids 1–52), followed by GFP, and then HDEL. C-terminal GFP and mCherry fusions were constructed as described previously (Lee et al., 2013; Longtine et al., 1998) and verified using PCR.

### Live-cell imaging

Cells were grown to mid-log phase in imaging medium (synthetic minimal medium supplemented with 20 µg/ml adenine, uracil, L-histadine and L-methionine; 30 µg/ml L-leucine and L-lysine; and 2% glucose; all purchased from Sigma-Aldrich) were immobilized on coverslips coated with 0.2 mg/ml concanavalin A and were imaged in imaging media.

Epifluorescence microscopy was conducted using a Nikon Eclipse Ti inverted microscope with a Nikon 100×1.4-NA Plan Apo VC oil-immersion objective and an Andor Neo 5.5 sCMOS camera. A Lumencore Spectra X LED light source with an FF-493/574-Di01 dual-pass dichroic mirror and FF01-512/630-25 dual-pass emission filters (Semrock) was used for two-color imaging of GFP and mCherry channels. This setup was controlled by Nikon Elements software ﻿and maintained at 25°C by an environmental chamber (In Vivo Scientific).

To study organelle inheritance events relative to time of bud emergence, cells were first imaged under bright field for various times to capture the moment bud emergence occurred. Immediately afterwards, *Z*-stacks with nine slices separated by 0.5 µm were collected using epifluorescence microscopy (time series duration and sampling frequency specified in figure legends) to monitor inheritance of fluorescently labelled organelles.

Image visualization was carried out with Fiji software (National Institutes of Health). For figure panels, cells were cropped, background signal was uniformly subtracted, and photobleaching was corrected using a custom Fiji macro (available from the authors upon request). Figures were then assembled in Adobe Illustrator 2019.

### Hydroxyurea and alpha factor experiments

Appropriate working concentrations of hydroxyurea and α-factor were determined empirically (Fig. S1A,B) and were generally in line with concentrations used previously (Amberg et al., 2005).

Hydroxyurea was purchased from Sigma-Aldrich. For single arrest experiments, cells were adhered to coverslips with concanavalin A and treated with 500 µl of 300 mM hydroxyurea in imaging medium for 3 h. Cells were then imaged using epifluorescence microscopy.

Alpha factor was synthesized by David King (University of California, Berkeley, USA) and stored as a stock at 10 mg/ml in 0.1 M sodium acetate buffer (pH 5.2). Cells were adhered to coverslips with concanavalin A and submerged in 500 µl of 3 µM α-factor in imaging medium for 3 h. To release from the arrest, the imaging medium with α-factor was removed and new medium with 0.1 mg/ml Pronase E (Sigma P-6911) was added to inactivate any remaining α-factor. This process was repeated two or three times after which 1.5 ml of imaging medium with 300 mM hydroxyurea was added.

### Flow cytometry

Flow cytometry was performed essentially as in Bloom et al. (2018). Hydroxyurea-treated cells were pelleted, washed with water, and then fixed with 70% ethanol. The fixed cells were subsequently washed twice with Tris-EDTA (pH 8.0) containing 0.1% Tween 20, then treated with 0.25 mg/ml RNase A in 50 mM sodium citrate (pH 7.2) containing 0.1% Tween 20 (citrate buffer) at 37°C overnight. Proteinase K was added to a final concentration of 0.2 mg/ml, and the cells were incubated for an additional 2 h at 50°C, then pelleted, resuspended in citrate buffer, sonicated for 30 s to disaggregate the cells, and stained with SYBR Green I (Invitrogen) at 1× final concentration in citrate buffer. Fixed and stained cells were stored in the dark at 4°C until they were analyzed on a LSR II flow cytometer (BD Biosciences). Quantification was performed using FlowJo analysis software. Forward scatter and side scatter gates were drawn individually for each hydroxyurea concentration, because hydroxyurea treatment causes changes to cell size and shape.

### Data analysis

To measure fluorescence intensity of an organelle in the bud during inheritance, individual cells at the appropriate bud growth stages were cropped from time-lapse image series. Organelles in the cropped 3D time lapses were segmented using the Allen Cell Structure Segmenter (Allen Institute for Cell Science, Seattle, WA, USA) and stacks of segmented images at each time point were converted into summed projections. These time lapses were then analyzed using Fiji software (National Institutes of Health). Raw integrated fluorescence intensity was measured in manually drawn selections surrounding and encompassing the bud and normalized relative to the maximum total fluorescence for each time lapse. Time relative to bud emergence was calculated using the corresponding bright-field time-lapse series.

For all other image analysis, cells were first visualized in Fiji and background subtraction and photobleaching correction were applied as described in the ‘Live-cell imaging’ section. For the hydroxyurea experiments in Fig. 3, organelles in cells were characterized as ‘inherited’ if they were clearly present in the bud at the time of imaging, ‘not inherited’ if no organelles were seen in the bud, and ‘partially inherited’ if all organelles were either in the mother cell or crossing the bud neck. In characterizing the relative order of inheritance for two organelles, one organelle was considered inherited first if during the time-lapse the organelle entered the bud before the other organelle or if the organelle was present in the bud before the other organelle began to be segregated to the bud. The order was considered ‘indistinguishable’ if both organelles appeared to be inherited at the same time.

### Statistics and reproducibility of experiments

All data presented were replicated in at least three distinct experiments. Multiple cells from each replicate were analyzed and data from different days were pooled together because they were indistinguishable. The number of cells analyzed at each timepoint for Fig. 1 is displayed in Fig. S2, and the number of cells analyzed for the remainder of the results is shown in the figure legend.

Statistical analyses (Welch's ANOVA test followed by Games–Howell posthoc test) were performed in Python using the Pingouin statistical package (Vallat, 2018).

## Supplementary Material

Supplementary information

Reviewer comments
