# Supplementary material for: A preferred sequence for organelle inheritance during polarized cell growth
Source: J Cell Sci. 2021 Nov 2;134(21):jcs258856. doi: 10.1242/jcs.258856 (PMC8627559; doi:10.1242/jcs.258856)
Supplement: Supplementary information [file joces-134-258856-s1.pdf]

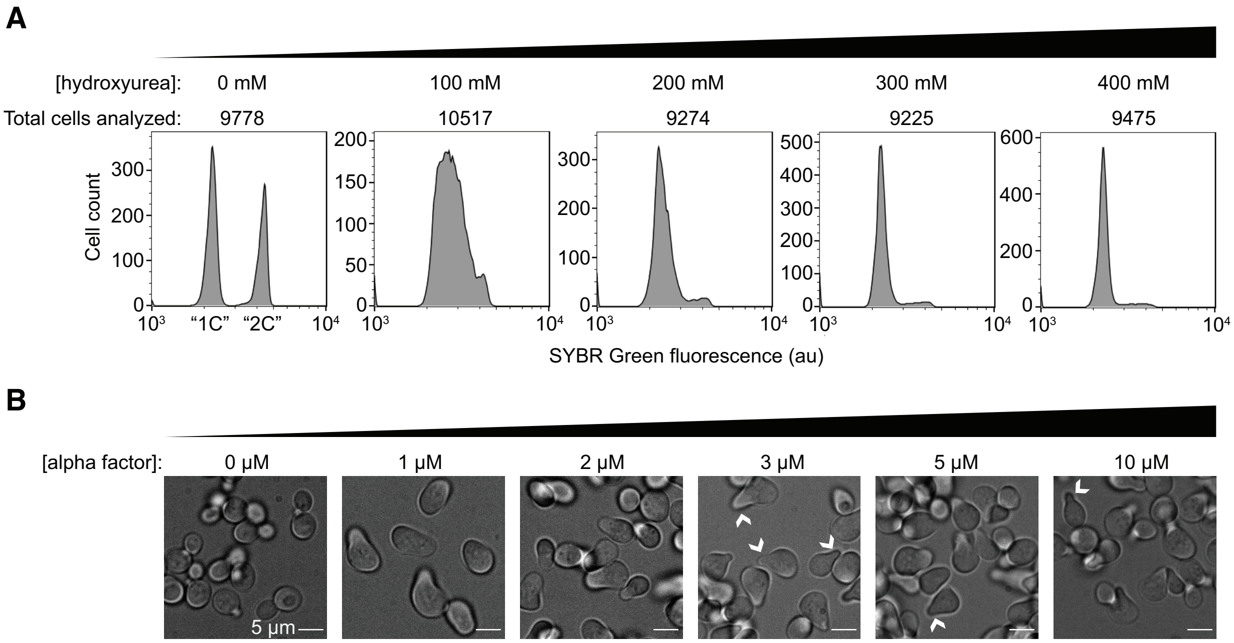

Fig. S1. Titration of hydroxyurea and alpha factor

(A) Effects of hydroxyurea on cells of our background. Cells were treated with hydroxyurea for five hours at the indicated concentration at room temperature in order to accentuate and saturate differences in the population of cells with replicated DNA ("2C"). They were then fixed, stained with SYBR Green to label DNA, and analyzed by flow cytometry. (B) Effects of alpha factor on matA cells of our background. Cells were submerged in alpha factor for four hours at the indicated concentration and imaged in brightfield. The effective alpha factor concentration was determined as the concentration under which most cells displayed well-defined mating projections (white chevrons).

Journal of Cell Science • Supplementary information

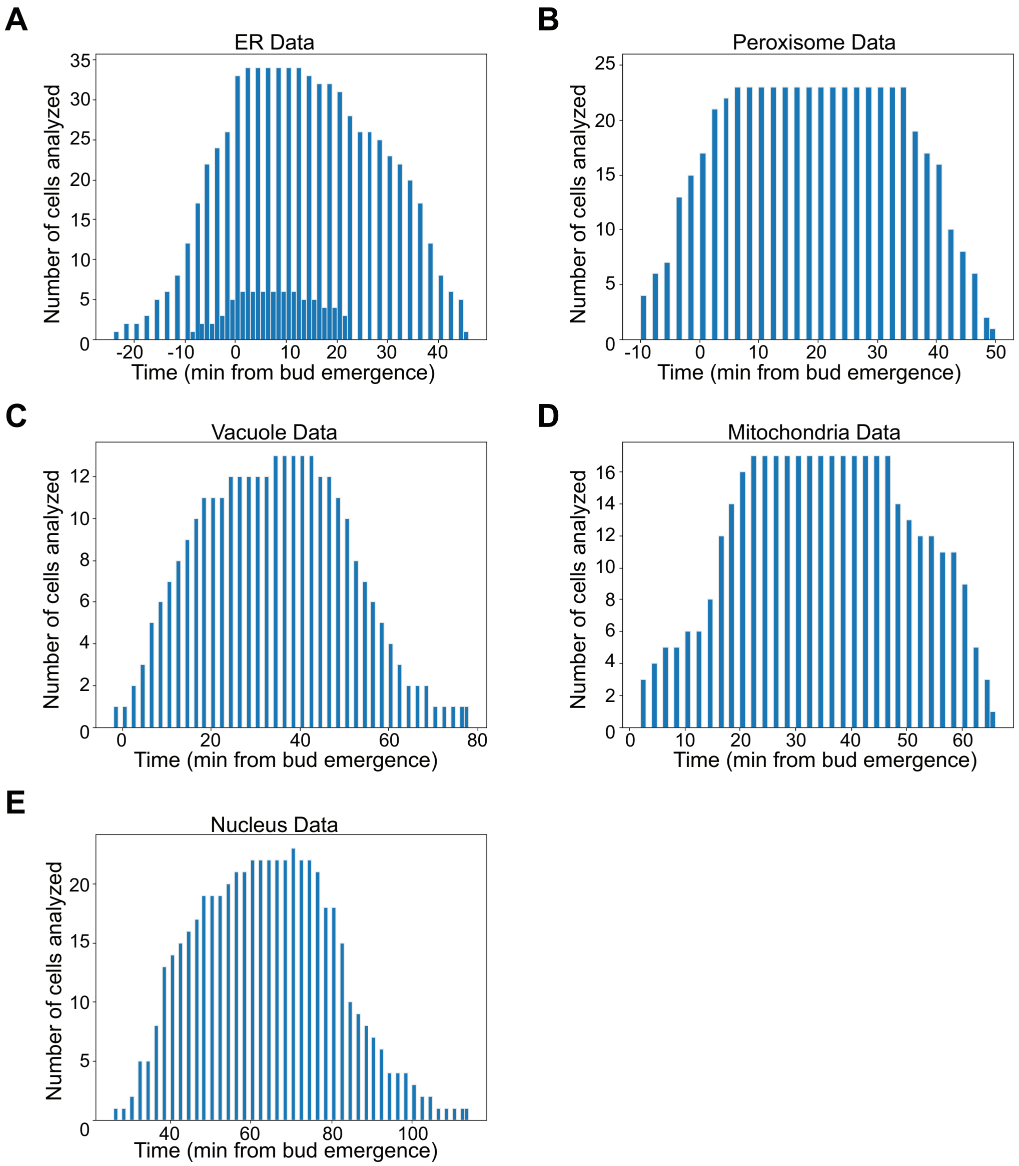

Fig. S2. Distributions of data points used in graphing organelle inheritance
Histograms depicting the number of cells analyzed per time point past bud emergence used to plot normalized bud fluorescence in Figure 1F. Data from cells labelling the ER (A), peroxisomes (B), vacuoles (C), mitochondria (D), and nucleus (E) are shown in separate panels.

Journal of Cell Science • Supplementary information

Table S1. Strains used in this study

Name	Genotype	Source
DDY1102	*MATa/MATα his3-Δ200/his3-Δ200, leu2-3, 112/leu2-3, 112, ura3-52/ura3-52, ade2-1/ADE2, lys2-801/LYS2*	Drubin laboratory collection
DDY5792	*MATα his3-Δ200, leu2-3, 112, ura3-52, Myo1-mCherry::KanMx, tpi1::pRS305-KAR2-GFP-HDEL*	This study
DDY5793	*MATα his3-Δ200, leu2-3, 112, ura3-52, Myo1-mCherry::KanMx, Pex3-GFP::HIS*	This study
DDY5794	*MATα his3-Δ200, leu2-3, 112, ura3-52, Myo1-mCherry::KanMx, Vph1-GFP::HIS*	This study
DDY5795	*MATα his3-Δ200, leu2-3, 112, ura3-52, Myo1-mCherry::KanMx, Cit1-GFP::HIS*	This study
DDY5796	*MATα his3-Δ200, leu2-3, 112, ura3-52, Myo1-mCherry::KanMx, Nup59-GFP::HIS*	This study
DDY5797	*MATα his3-Δ200, leu2-3, 112, ura3-52, Pex3-mCherry::KanMx, tpi1::pRS305-KAR2-GFP-HDEL*	This study
DDY5798	*MATα his3-Δ200, leu2-3, 112, ura3-52, Cit1-mCherry::KanMx, Vph1-GFP::HIS*	This study
DDY5799	*MATa his3-Δ200, leu2-3, 112, ura3-52, Pex3-mCherry::KanMx, tpi1::pRS305-KAR2-GFP-HDEL*	This study
DDY5800	*MATa his3-Δ200, leu2-3, 112, ura3-52, Cit1-mCherry::KanMx, Pex3-GFP::HIS*	This study
DDY5801	*MATa his3-Δ200, leu2-3, 112, ura3-52, Cit1-mCherry::KanMx, Vph1-GFP::HIS*	This study

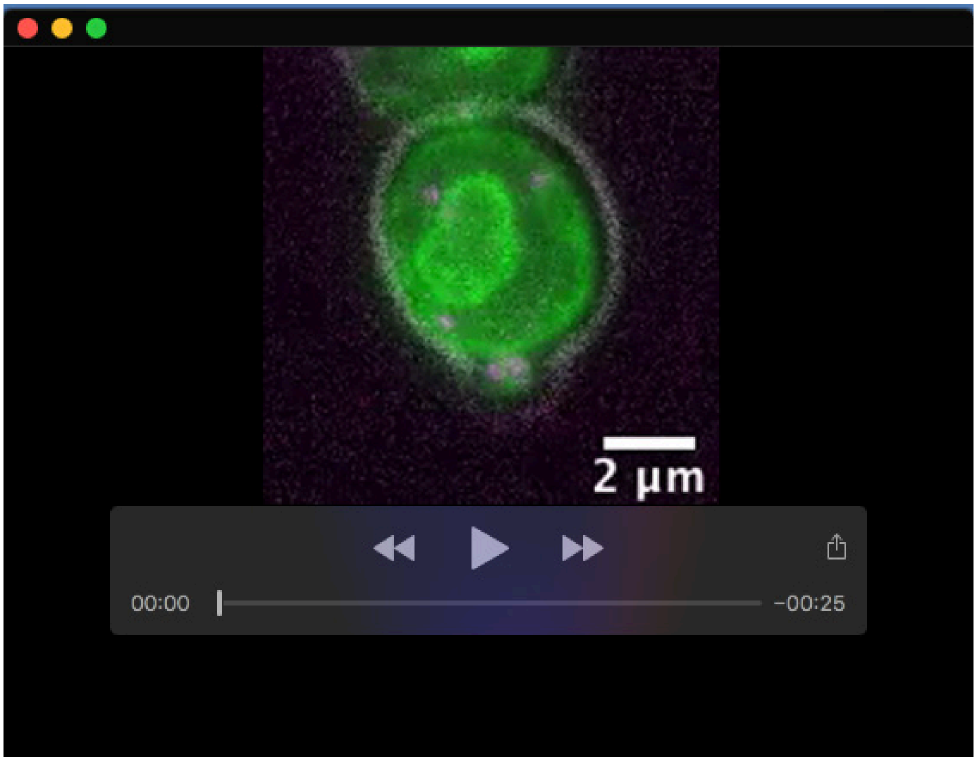

Movie 1. Inheritance of ER and peroxisomes into emerging buds
Maximum intensity projection movie from 3D time lapse epifluorescence imaging of a cell expressing a GFP-HDEL ER marker (green) and endogenously expressing a Pex3-mCherry peroxisome marker (magenta). The cell outline from bright field imaging is in gray. The movie is 26 frames long with 1 minute separating each frame, played back at 15 frames per second.

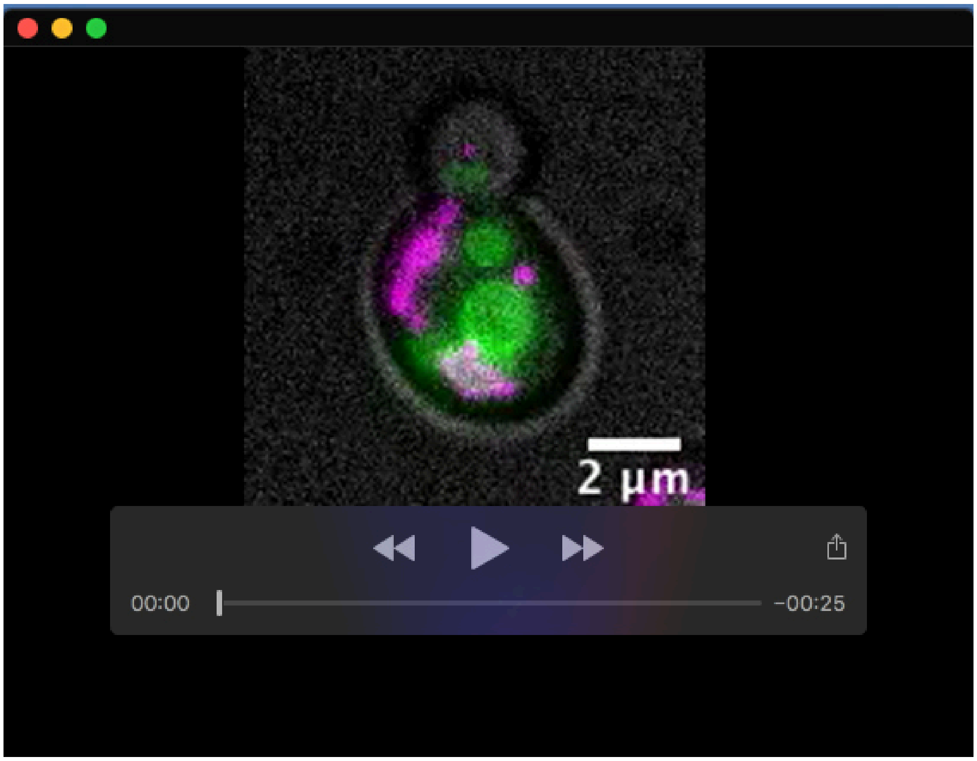

Movie 2. Inheritance of vacuoles and mitochondria into small buds
Maximum intensity projection movie from 3D time lapse epifluorescence imaging of a cell endogenously expressing a Vph1-GFP vacuole marker (green) and a Cit1-mCherry mitochondrial marker (magenta). The cell outline from bright field imaging is in gray. The movie is 26 frames long with 1 minute separating each frame, played back at 15 frames per second.

Journal of Cell Science • Supplementary information

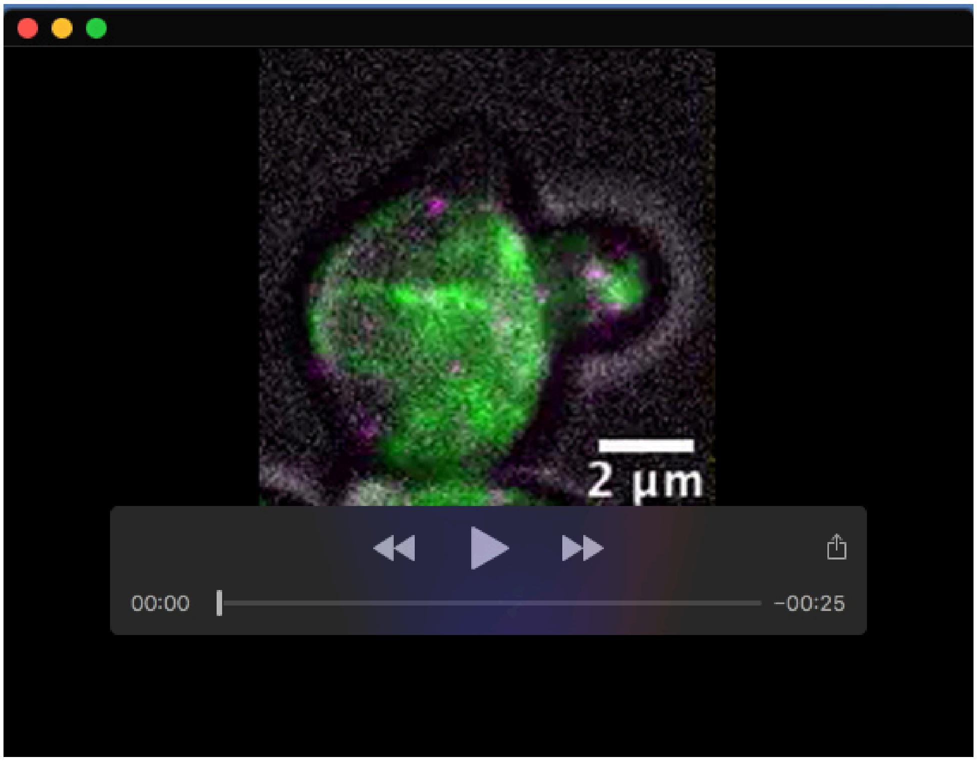

Movie 3. Inheritance of ER and peroxisomes in hydroxyurea-treated cells
Maximum intensity projection movie from 3D time lapse epifluorescence imaging of a cell expressing a GFP-HDEL ER marker (green) and endogenously expressing a Pex3-mCherry peroxisome marker (magenta), treated with hydroxyurea to prevent S-phase completion. The cell outline from bright field imaging is in gray. The movie is 23 frames long with 2 minute separating each frame, played back at 15 frames per second.

Journal of Cell Science • Supplementary information

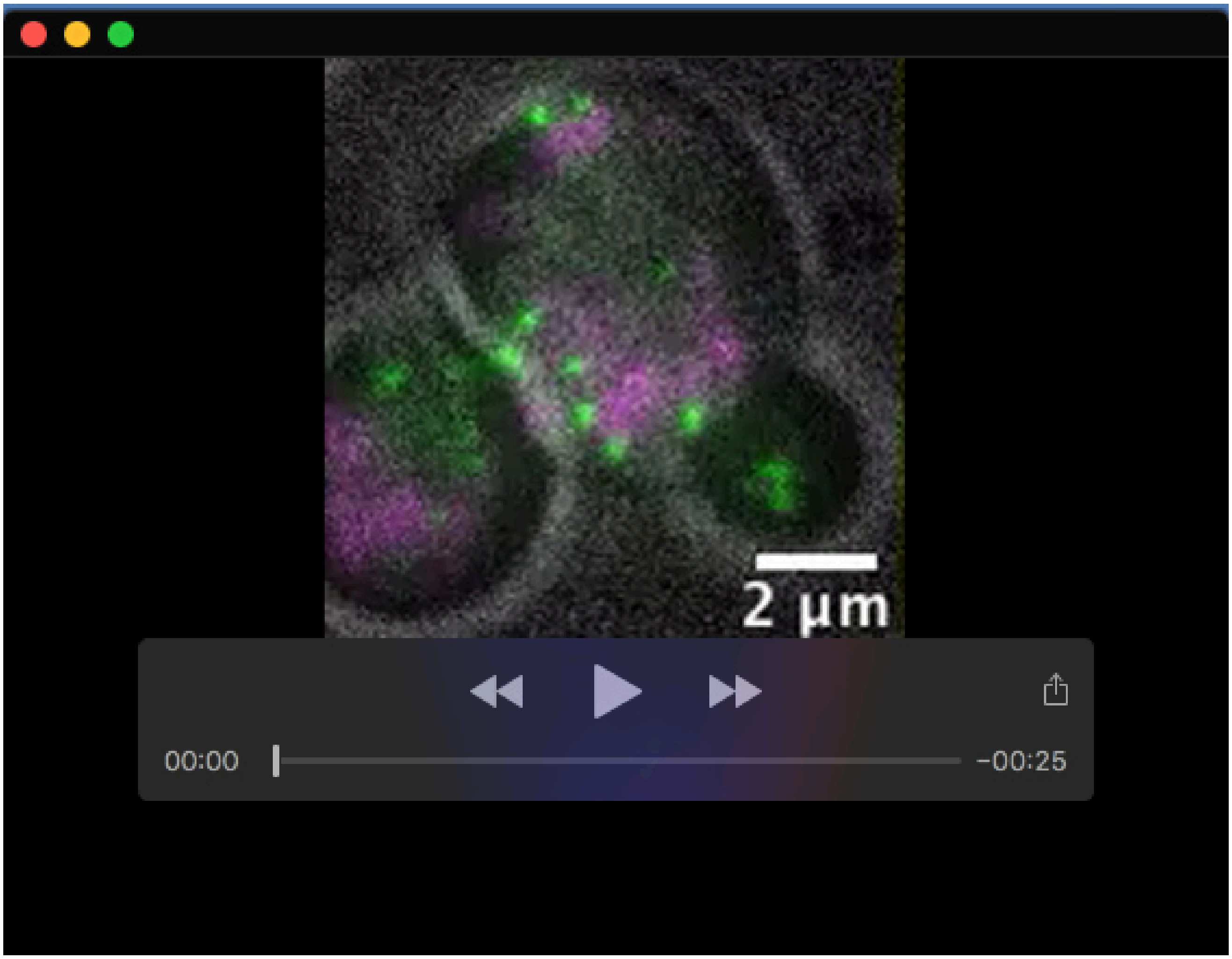

Movie 4. Inheritance of peroxisomes and mitochondria in hydroxyurea-treated cells
Maximum intensity projection movie from 3D time lapse epifluorescence imaging of a cell endogenously expressing a Pex3-GFP peroxisome marker (green) and a Cit1-mCherry mitochondrial marker (magenta), treated with hydroxyurea to prevent S-phase completion. The cell outline from bright field imaging is in gray. The movie is 23 frames long with 2 minute separating each frame, played back at 15 frames per second.

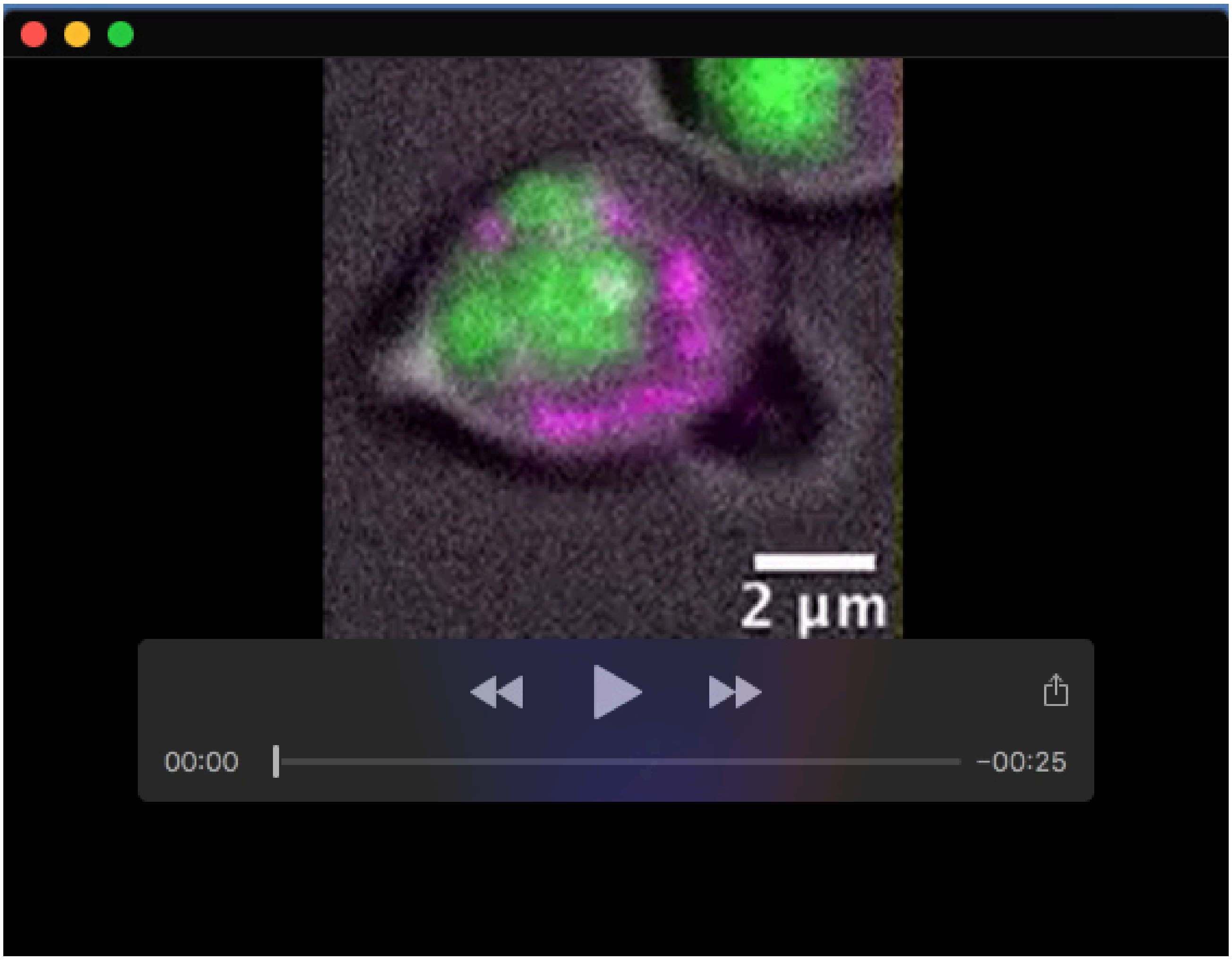

Movie 5. Inheritance of vacuoles and mitochondria in hydroxyurea-treated cells
Maximum intensity projection movie from 3D time lapse epifluorescence imaging of a cell endogenously expressing a Vph1-GFP vacuole marker (green) and a Cit1-mCherry mitochondrial marker (magenta), treated with hydroxyurea to prevent S-phase completion. The cell outline from bright field imaging is in gray. The movie is 23 frames long with 2 minute separating each frame, played back at 15 frames per second.
