# Supplementary material for: A preferred sequence for organelle inheritance during polarized cell growth
Source: J Cell Sci. 2021 Nov 2;134(21):jcs258856. doi: 10.1242/jcs.258856 (PMC8627559; doi:10.1242/jcs.258856)
Supplement: Reviewer comments [file joces-134-258856_review_history.pdf]

A preferred sequence for organelle inheritance during polarized cell growth

Kathryn W. Li, Michelle S. Lu, Yuichiro Iwamoto, David G. Drubin and Ross T. A. Pedersen
DOI: 10.1242/jcs.258856

Editor: Jennifer Lippincott-Schwartz

Review timeline

Original submission:	29 April 2021
Editorial decision:	21 June 2021
First revision received:	23 August 2021
Accepted:	27 September 2021

Original submission

First decision letter

MS ID#: JOCES/2021/258856

MS TITLE: An organelle inheritance pathway during polarized cell growth

AUTHORS: David G Drubin, Kathryn W Li, Ross TA Pedersen, and Michelle S Lu
ARTICLE TYPE: Short Report
We have now reached a decision on the above manuscript.

To see the reviewers' reports and a copy of this decision letter, please go to: https://submit-jcs.biologists.org and click on the 'Manuscripts with Decisions' queue in the Author Area. (Corresponding author only has access to reviews.)

As you will see, the reviewers raise a number of substantial criticisms that prevent me from accepting the paper at this stage. They suggest, however, that a revised version might prove acceptable, if you can address their concerns. If you think that you can deal satisfactorily with the criticisms on revision, I would be pleased to see a revised manuscript. We would then return it to the reviewers.

We are aware that you may be experiencing disruption to the normal running of your lab that makes experimental revisions challenging. If it would be helpful, we encourage you to contact us to discuss your revision in greater detail. Please send us a point-by-point response indicating where you are able to address concerns raised (either experimentally or by changes to the text) and where you will not be able to do so within the normal timeframe of a revision. We will then provide further guidance. Please also note that we are happy to extend revision timeframes as necessary.

Please ensure that you clearly highlight all changes made in the revised manuscript. Please avoid using 'Tracked changes' in Word files as these are lost in PDF conversion.

I should be grateful if you would also provide a point-by-point response detailing how you have dealt with the points raised by the reviewers in the 'Response to Reviewers' box. Please attend to all of the reviewers' comments. If you do not agree with any of their criticisms or suggestions please explain clearly why this is so.

© 2021. Published by The Company of Biologists under the terms of the Creative Commons Attribution License (https://creativecommons.org/licenses/by/4.0/).

Reviewer 1

Advance summary and potential significance to field

Using multiple fluorescent tagged proteins, the authors track the inheritance of five organelles in the simple model budding yeast. The relative temporal ordering of four of the five organelles was not previously well described. Considering the method of the inheritance for those four organelles is thought to be similar (myosin V-dependent transport along actin cables), it is novel the authors demonstrate there is a consistent temporal ordering of their inheritance, and that broad aspects of the temporal ordering of these organelles does not depend on transition of the cell cycle from G1 to S phase. This work demonstrates there exists some non-cell cycle-dependent mechanism(s) that orders organelle inheritance, although my enthusiasm for this is tempered by the fact that the nature of this mechanism is far from clear.

Comments for the author

The work presented is straightforward and clear. However, I believe the authors overstate the significance of their conclusion with the proposed title, "An organelle inheritance pathway during polarized cell growth". The word "pathway" implies a defined interdependence of events. The authors show there is a stereotypical order in timing, but no evidence there is an interdependence in these events or any commonality in the mechanisms that lead to this ordering, which I would consider a minimal requirement to describe this as a "pathway". The authors themselves even back off the conclusion they have evidence of a single "organelle inheritance pathway" at the end of the Introduction, where they invoke the likelihood of "interdependent translocation or signaling pathways". At minimum, the title should be revised.

I would also strongly recommend the authors consider revising their text as regards nuclear inheritance. Their observations that the nucleus/perinuclear ER is inherited later than the other components is treated on an equal footing as the other organelles. The relatively late timing of nuclear inheritance is well-known, and its dependence on a different system for inheritance (i.e. microtubules, and particularly late dynein-dependent sliding of microtubules along the cortex) makes this result unsurprising. Rather, I would recommend the authors highlight the commonality in mechanism for how the other four organelles are inherited, which would make one naively predict their inheritance would be in random order or simultaneous.

A final suggestion, the text that initially refers to the Movie 1 is somewhat confusing. Line 105 describes Pex3 being visualized via GFP tag, and line 119 refers to those results, including Movie 1. However, the GFP channel in Movie 1 is GFP-HDEL/Pex3-mCherry. The reader will eventually catch on (after reading the Movie 1 legend) but I was so prepped to look at the green channel, I didn’t realize I should instead be focusing on small magenta puncta, and I was assuming there had been a mix-up in the movies.

Reviewer 2

Advance summary and potential significance to field

Multiple studies have addressed the dynamics of organelle inheritance, however in most studies specific organelles have been studied individually. This manuscript reports on the relative timing of organelle inheritance by analyzing the inheritance of five yeast organelles. The authors find that the timing of inheritance occurs at specific times. The authors further suggest that organelle inheritance occurs even when cells are arrested in S-phase of the cell-cycle.

Comments for the author

1. In Figure 4 where cells are arrested with alpha-factor and released into hydroxy-urea, the authors need to demonstrate that the cells are in fact arrested in S-phase. An alternative explanation of the data is that the hydroxyurea did not cause a block. One way to test whether cells are arrested in S-phase is to perform FACs analysis on the cultures.

2. The authors used two different concentrations of hydroxyurea. Is there a specific reason why?

© 2021. Published by The Company of Biologists under the terms of the Creative Commons Attribution License (https://creativecommons.org/licenses/by/4.0/).

3. In figure 3 the authors should indicate the number of times the experiment was performed, as well as the number of cells that were scored in each category. In addition, they should perform a statistical analyses of the data.

4. In the abstract, the authors conclude that “Thus, organelle inheritance follows a preferred order during polarized cell division, but it is not controlled exclusively by cell cycle signaling.” This statement is too general. In this manuscript the authors provide evidence that organelle inheritance does not required DNA synthesis in S-phase, yet they observe that each organelle moves into the bud at a specific bud size. This indicates that organelle inheritance may be linked with some aspect of the cell cycle, but not DNA synthesis in S-phase.

5. Several papers have reported that hydroxyurea treatment does not inhibit organelle transport to the bud (Fagarasanu et al., 2005 PMID 15928207; Loewen et al., 2007 PMID 17984322; Kraft and Lackner, 2017 PMID 28835466). These papers should be cited.

6.The Golgi is also inherited (Rossanese et al., 2001 PMID 11285273; Arai et al., 2008 PMID 18595704). The authors should explain why they excluded this organelle.

7. The authors should compare their results with a previous study that reported that the timing of mitochondria inheritance and vacuole inheritance are similar (Eves et al., 2012 PMID 22753895).

8. The authors offer several suggestions of possible mechanisms that contribute to the timing of the inheritance of the organelles. They may also want to consider specific limitations, for example the size of the opening at the mother-bud neck.
Or comment on what is known about the regulation of Myo2 or Myo4 and/or their organelle-specific adaptors.

7. The authors should include more information of live-cell imaging (acquisition speed, how many z-stack for maximum projection, how to maintain cell condition after immobilized to coverslips during live-cell imaging).

Reviewer 3

Advance summary and potential significance to field

In the manuscript, Li et al. investigated organelle's segregation behaviors in budding yeasts during asymmetric cell division. The authors described a distinct organellar segregation pattern into daughter cells, which is independent on the progression of S-phase DNA synthesis under a synthetic minimum medium regime. Using 3D imaging approach with cell cycle modulation the study presents interesting behavioral observations from five key organelles (i.e., peroxisome, ER, mitochondrion, vacuole, and nucleus) during cell division. However, the findings are mainly descriptive and not based on concurrent and integrative assessments for multiple organelles. The study failed to provide strong mechanistic insights on how and which cellular programs control organelle inheritance with such specific temporal ordering. To fulfill the journal’s standard, the current study surely needs more extensive development, and for this reason, this reviewer would state that the manuscript is insufficient for publication.

Comments for the author

One potential mechanism that the authors can consider to interrogate further with their experimental system is to check if organellar contact governed by various tether complexes (e.g., ERMES, vCLAMP, NVJ, and PM-ER, etc.) has any impact on the regulation of both relative ordering and absolute timing of organelle inheritance. Given the fact that cells become very susceptible to cytotoxic stress during cytokinesis, understanding cellular programs that determine the absolute timing (or duration) of organellar segregation would significantly advance the fields of cell science.

© 2021. Published by The Company of Biologists under the terms of the Creative Commons Attribution License (https://creativecommons.org/licenses/by/4.0/).

First revision

Author response to reviewers' comments

Reviewer 1 Advance Summary and Potential Significance to Field:
Using multiple fluorescent tagged proteins, the authors track the inheritance of five organelles in the simple model budding yeast. The relative temporal ordering of four of the five organelles was not previously well described. Considering the method of the inheritance for those four organelles is thought to be similar (myosin V-dependent transport along actin cables), it is novel the authors demonstrate there is a consistent temporal ordering of their inheritance, and that broad aspects of the temporal ordering of these organelles does not depend on transition of the cell cycle from G1 to S phase. This work demonstrates there exists some non-cell cycle-dependent mechanism(s) that orders organelle inheritance, although my enthusiasm for this is tempered by the fact that the nature of this mechanism is far from clear.

We thank this reviewer for the overall positive assessment of our manuscript. While we recognize that the current work does not fully reveal the mechanism for the observed phenomena, we believe that these kinds of "descriptive" studies are the backbone of cell biology. One must describe a process before the mechanism can be elucidated.
Indeed, many "classic" papers in cell biology are "descriptive," but are nonetheless highly cited because all subsequent mechanistic work cites the original study. A good example is Tilney and Portnoy's 1989 JCB paper describing *Listeria monocytogenes* actin tails, which, like our work, is mostly descriptive, chronicling the succession of events during *Listeria* cell invasion. The most "mechanistic" experiments in the paper rely on straightforward pharmacological experiments, again paralleling our study. The Tilney and Portnoy paper has been cited nearly 1500 times.

Reviewer 1 Comments for the Author:
The work presented is straightforward and clear. However, I believe the authors overstate the significance of their conclusion with the proposed title, "An organelle inheritance pathway during polarized cell growth". The word "pathway" implies a defined interdependence of events. The authors show there is a stereotypical order in timing, but no evidence there is an interdependence in these events or any commonality in the mechanisms that lead to this ordering, which I would consider a minimal requirement to describe this as a "pathway". The authors themselves even back off the conclusion they have evidence of a single "organelle inheritance pathway" at the end of the Introduction, where they invoke the likelihood of "interdependent translocation or signaling pathways". At minimum, the title should be revised.

The reviewer raises a good point here and we thank them for this criticism. At the very least, we should be internally consistent. Per this recommendation, we have edited the title to "A preferred sequence for organelle inheritance during polarized cell growth."

I would also strongly recommend the authors consider revising their text as regards nuclear inheritance. Their observations that the nucleus/perinuclear ER is inherited later than the other components is treated on an equal footing as the other organelles. The relatively late timing of nuclear inheritance is well-known, and its dependence on a different system for inheritance (i.e. microtubules, and particularly late dynein- dependent sliding of microtubules along the cortex) makes this result unsurprising.

Rather, I would recommend the authors highlight the commonality in mechanism for how the other four organelles are inherited, which would make one naively predict their inheritance would be in random order or simultaneous.

We thank the reviewer for this suggestion. We have edited the text in several places, namely Introduction paragraphs 3 and 4 and Results/Discussion paragraphs 3 and 6, in an attempt to deemphasize our nuclear inheritance results and instead emphasize how noteworthy it is that the other organelles studied are inherited in a preferred sequence, despite the fact that their inheritance mechanisms share considerable commonality.

© 2021. Published by The Company of Biologists under the terms of the Creative Commons Attribution License (https://creativecommons.org/licenses/by/4.0/).

A final suggestion, the text that initially refers to the Movie 1 is somewhat confusing. Line 105 describes Pex3 being visualized via GFP tag, and line 119 refers to those results, including Movie 1. However, the GFP channel in Movie 1 is GFP-HDEL/Pex3- mCherry. The reader will eventually catch on (after reading the Movie 1 legend) but I was so prepped to look at the green channel, I didn't realize I should instead be focusing on small magenta puncta, and I was assuming there had been a mix-up in the movies.

We referred to Movie 1 in line 119 of the original text to illustrate the point made in the preceding sentence: "Peroxisomes are the most dynamic of the organelles that we imaged, and they became particularly difficult to track as the growing bud got bigger, allowing them more space to dynamically occupy." However, we now realize that this was confusing, because, as the reviewer points out, at that point in the manuscript we had not yet introduced the GFP-HDEL/Pex3-mCherry yeast strain. To avoid similar confusion for future readers, we have removed the reference to Movie 1 in the location at question.

Reviewer 2 Advance Summary and Potential Significance to Field:
Multiple studies have addressed the dynamics of organelle inheritance, however in most studies specific organelles have been studied individually. This manuscript reports on the relative timing of organelle inheritance by analyzing the inheritance of five yeast organelles. The authors find that the timing of inheritance occurs at specific times. The authors further suggest that organelle inheritance occurs even when cells are arrested in S-phase of the cell-cycle.

Reviewer 2 Comments for the Author:

1. In Figure 4 where cells are arrested with alpha-factor and released into hydroxy-urea, the authors need to demonstrate that the cells are in fact arrested in S-phase. An alternative explanation of the data is that the hydroxyurea did not cause a block. One way to test whether cells are arrested in S-phase is to perform FACs analysis on the cultures.

We suspect that the reviewer's doubt that the cells shown in Figure 4 are arrested by hydroxyurea stems from a typographical error made on our part, and we have now corrected the error. We used 200 mM and 300 mM hydroxyurea, but in preparing our manuscript erroneously reported using 200 and 300 µM. We apologize for causing this confusion with our typo.

To demonstrate that our hydroxyurea treatment effectively blocked S-phase, we have now performed flow cytometry on cells stained with the DNA dye SYBR Green.
Treatment with 200-400 mM hydroxyurea effectively eliminated the population of cells with replicated DNA (i.e. 2C DNA content). These data are now presented in supplemental figure S1A.

2. The authors used two different concentrations of hydroxyurea. Is there a specific reason why?

We had originally intended to repeat the experiments summarized in Figure 3 with the higher hydroxyurea concentration for the sake of consistency, but lab closure due to the pandemic and the departure of authors from the lab foiled our plans.

Nevertheless, we have now managed to repeat the experiments in Figure 3 at the higher hydroxyurea concentration, bringing consistency to our manuscript. We have replaced all panels of Figure 3 with panels from the new experiment. In selecting which micrographs to include as representative examples, we took care to choose some cells where the bud appears unusually long, a hallmark of hydroxyurea arrest that becomes particularly pronounced at later time points (see, for example, Fagarasanu et al., 2005 PMID 15928207, Figure 6), which we hope will reassure readers that the hydroxyurea treatment was effective.

3. In figure 3 the authors should indicate the number of times the experiment was performed, as well as the number of cells that were scored in each category. In addition, they should perform a statistical analyses of the data.

The number of cells analyzed for each experiment and the number of replicates is now described

© 2021. Published by The Company of Biologists under the terms of the Creative Commons Attribution License (https://creativecommons.org/licenses/by/4.0/).

in figure legends throughout the manuscript. We have also added numbers to our stacked bar charts indicating the actual percentages, not only in Figure 3, but throughout the manuscript. With the number of cells analyzed and actual percentage values now transparently available, readers can quickly and easily multiply to arrive at the number of cells scored in each category.

We weren't sure what the reviewer had in mind in terms of statistical analysis for figure 3, so we did a series of chi-squared tests. This experiment does not compare the inheritance of the different organelles, so a statistical test that asks whether the organelles are inherited differently does not seem appropriate. We don't understand what is being asked of us specifically. In a good-faith effort to address this reviewer's concern, we performed a two-tailed chi-squared test for the inheritance of each organelle with the null hypothesis being that the organelles have a likelihood of being found in the mother (not inherited), bud neck (partially inherited) or bud (inherited) proportional to the relative areas of these regions. The results of these chi-squared tests are now presented in lines 189-194 of the text.

4. In the abstract, the authors conclude that "Thus, organelle inheritance follows a preferred order during polarized cell division, but it is not controlled exclusively by cell cycle signaling." This statement is too general. In this manuscript the authors provide evidence that organelle inheritance does not required DNA synthesis in S-phase, yet they observe that each organelle moves into the bud at a specific bud size. This indicates that organelle inheritance may be linked with some aspect of the cell cycle, but not DNA synthesis in S-phase.

This is an instructive piece of criticism, and we now recognize that we did not write precisely enough about our cell cycle experiments. We have made edits to the Abstract and throughout the manuscript to communicate that the actual effect of our drug treatments was prevention of S-phase completion.

5. Several papers have reported that hydroxyurea treatment does not inhibit organelle transport to the bud (Fagarasanu et al., 2005 PMID 15928207; Loewen et al., 2007 PMID 17984322; Kraft and Lackner, 2017 PMID 28835466). These papers should be cited.

We thank the reviewer for bringing these results to our attention. We have added citations to Fagarasanu et al. and Loewen et al. We cited Yang et al., 1999 (PMID 10531006) as an example of a study demonstrating that mitochondria are inherited under hydroxyurea treatment, because it predates Kraft and Lackner 2017 and presents the data more directly. Kraft and Lackner is nevertheless cited in our manuscript elsewhere. In fact, we dedicate a paragraph of the Introduction almost exclusively to discussing Kraft and Lackner 2017, as it is a major motivator for this study.

6. The Golgi is also inherited (Rossanese et al., 2001 PMID 11285273; Arai et al., 2008 PMID 18595704). The authors should explain why they excluded this organelle.

We have added a sentence citing these papers and explaining why this organelle was excluded from our study to the first paragraph of the Results and Discussion section. In short, we excluded the Golgi apparatus from our study because it can be generated *de novo* as well as being inherited.

7. The authors should compare their results with a previous study that reported that the timing of mitochondria inheritance and vacuole inheritance are similar (Eves et al., 2012 PMID 22753895).

We have added two sentences comparing our results with those of Eves et al. and offering one potential explanation for the discrepancy to the manuscript, immediately following the reference to Fig. 2B. We mention as a possible explanation for this discrepancy our use of 3D time lapse imaging, as opposed to imaging only in the medial focal plane of the cells, which was used in that previous study.

8. The authors offer several suggestions of possible mechanisms that contribute to the timing of the inheritance of the organelles. They may also want to consider specific limitations, for example the size of the opening at the mother-bud neck. Or comment on what is known about

© 2021. Published by The Company of Biologists under the terms of the Creative Commons Attribution License (https://creativecommons.org/licenses/by/4.0/).

the regulation of Myo2 or Myo4 and/or their organelle-specific adaptors.

We thank the reviewer for this recommendation. We agree that readers may be interested in further discussion of possible mechanisms governing the order of organelle inheritance. We have added additional discussion of these kinds of limitations to the final paragraphs of the Results and Discussion section. Due to word count limitations, we endeavored to keep the text that we added brief.

7. The authors should include more information of live-cell imaging (acquisition speed, how many z-stack for maximum projection, how to maintain cell condition after immobilized to coverslips during live-cell imaging).

We apologize for this oversight. The requested information has been added, with parameters that were constant for all experiments described in the Materials and Methods section, and other details included in figure legends where appropriate.

Reviewer 3 Advance Summary and Potential Significance to Field:
In the manuscript, Li et al. investigated organelle's segregation behaviors in budding yeasts during asymmetric cell division. The authors described a distinct organellar segregation pattern into daughter cells, which is independent on the progression of S- phase DNA synthesis under a synthetic minimum medium regime. Using 3D imaging approach with cell cycle modulation, the study presents interesting behavioral observations from five key organelles (i.e., peroxisome, ER, mitochondrion, vacuole, and nucleus) during cell division. However, the findings are mainly descriptive and not based on concurrent and integrative assessments for multiple organelles. The study failed to provide strong mechanistic insights on how and which cellular programs control organelle inheritance with such specific temporal ordering. To fulfill the journal's standard, the current study surely needs more extensive development, and for this reason, this reviewer would state that the manuscript is insufficient for publication.

Reviewer 3 Comments for the Author:
One potential mechanism that the authors can consider to interrogate further with their experimental system is to check if organellar contact governed by various tether complexes (e.g., ERMES, vCLAMP, NVJ, and PM-ER, etc.) has any impact on the regulation of both relative ordering and absolute timing of organelle inheritance. Given the fact that cells become very susceptible to cytotoxic stress during cytokinesis, understanding cellular programs that determine the absolute timing (or duration) of organellar segregation would significantly advance the fields of cell science.

We are glad that this reviewer found our observations interesting and appreciate the criticism of our work, but we respectfully disagree with the reviewer's negative assessment of studies such as ours that are mainly observational. While we recognize that the current work does not fully reveal the mechanism for the observed phenomena, we believe that "descriptive" studies are the backbone of cell biology. One must describe a process before the mechanism can be elucidated. Indeed, many "classic" papers in cell biology are "descriptive," but are nonetheless highly cited because all subsequent mechanistic work cites the original study. A good example is Tilney and Portnoy's 1989 JCB paper describing *Listeria monocytogenes* actin tails, which, like our work, is mostly descriptive, chronicling the succession of events during *Listeria* cell invasion. The most "mechanistic" experiments in the paper rely on straightforward pharmacological experiments, again paralleling our study. The Tilney and Portnoy paper has been cited nearly 1500 times.

Second decision letter

MS ID#: JOCES/2021/258856

MS TITLE: A preferred sequence for organelle inheritance during polarized cell growth

AUTHORS: David G Drubin, Kathryn W Li, Ross TA Pedersen, Michelle S Lu, and Yuichiro Iwamoto

© 2021. Published by The Company of Biologists under the terms of the Creative Commons Attribution License (https://creativecommons.org/licenses/by/4.0/).

ARTICLE TYPE: Short Report

I am happy to tell you that your manuscript has been accepted for publication in Journal of Cell Science, pending standard ethics checks.

Reviewer 1

Advance summary and potential significance to field

Using multiple fluorescent tagged proteins, the authors track the inheritance of five organelles in the simple model budding yeast. The relative temporal ordering of four of the five organelles was not previously well described. Considering the method of the inheritance for those four organelles is thought to be similar (myosin V-dependent transport along actin cables), it is novel the authors demonstrate there is a consistent temporal ordering of their inheritance, and that broad aspects of the temporal ordering of these organelles does not depend on transition of the cell cycle from G1 to S phase. This work demonstrates there exists some non-cell cycle-dependent mechanism(s) that orders organelle inheritance.

Comments for the author

I am satisfied that the authors have addressed all of my previous comments.

Reviewer 2

Advance summary and potential significance to field

Multiple studies have addressed the dynamics of organelle inheritance, however in most studies specific organelles have been studied individually or in pairs. This manuscript reports on the relative timing of organelle inheritance by analyzing the inheritance of five yeast organelles. The authors find that for each organelle inheritance occurs at specific times. The authors further suggest that organelle inheritance occurs even when cells are arrested in S-phase of the cell-cycle. These observational studies will provide a basis for future mechanistic studies of how a single molecular motor coordinates the movement of multiple cargoes.
Moreover, these findings hint at the existence of cell-cycle pathways that are independent of DNA replication.

Comments for the author

The authors have adequately addressed each of the concerns raised in the original review.

© 2021. Published by The Company of Biologists under the terms of the Creative Commons Attribution License (https://creativecommons.org/licenses/by/4.0/).
